# On Higher Ground: How Well Can Dynamic Body Acceleration Determine Speed in Variable Terrain?

**DOI:** 10.1371/journal.pone.0050556

**Published:** 2012-11-30

**Authors:** Owen R. Bidder, Lama A. Qasem, Rory P. Wilson

**Affiliations:** Biological Sciences, College of Science, Swansea University, Swansea, United Kingdom; University of Arizona, United States of America

## Abstract

**Introduction:**

Animal travel speed is an ecologically significant parameter, with implications for the study of energetics and animal behaviour. It is also necessary for the calculation of animal paths by dead-reckoning. Dead-reckoning uses heading and speed to calculate an animal’s path through its environment on a fine scale. It is often used in aquatic environments, where transmission telemetry is difficult. However, its adoption for tracking terrestrial animals is limited by our ability to measure speed accurately on a fine scale. Recently, tri-axial accelerometers have shown promise for estimating speed, but their accuracy appears affected by changes in substrate and surface gradients. The purpose of the present study was to evaluate four metrics of acceleration; Overall dynamic body acceleration (ODBA), vectorial dynamic body acceleration (VDBA), acceleration peak frequency and acceleration peak amplitude, as proxies for speed over hard, soft and inclined surfaces, using humans as a model species.

**Results:**

A general linear model (GLM) showed a significant difference in the relationships between the metrics and speed depending on substrate or surface gradient. When the data from all surface types were considered together, VeDBA had the highest coefficient of determination.

**Conclusions:**

All of the metrics showed some variation in their relationship with speed according to the surface type. This indicates that changes in the substrate or surface gradient during locomotion by animals would produce errors in speed estimates, and also in dead-reckoned tracks if they were calculated from speeds based entirely on *a priori* calibrations. However, we describe a method by which the relationship between acceleration metrics and speed can be corrected *ad hoc*, until tracks accord with periodic ground truthed positions, obtained via a secondary means (e.g. VHF or GPS telemetry). In this way, dead-reckoning provides a means to obtain fine scale movement data for terrestrial animals, without the need for additional data on substrate or gradient.

## Introduction

Quantification of fine-scale animal movement is critical for understanding animal ecology because movement determines access to resources and helps avoid predation, which, ultimately, determines the success of individuals and modulates populations (see [1]). Attempts to monitor animal movements, however [2], [3], [4], [5], [6] are complicated when species are cryptic [7], secretive [8], move large distances [9], or simply operate in areas or at times where direct observations cannot be made [10], [11]. Specialist equipment such as night-vision systems, infra-red imagery and chemoluminescent tags have helped study nocturnal species [12], [13], [14], [15], [16] and ‘spool and thread’ methods have been used to study the movements of small mammals [17], [18], [19]. All such methods require significant field effort to implement.

Biotelemetric methods (see [20] for review) obviate the need for visual contact between researcher and study animal and have helped the study of movement ecology significantly, although most lack the spatial and temporal resolution to track fine scale animal movements which can be pivotal in understanding animal route choices.

To our knowledge, the only biotelemetric method that provides continuous, fine temporal scale positional data irrespective of radio- or acoustic links is dead-reckoning [21]. Dead-reckoning derives animal movement by reconstructing an animal’s travel path, using information on speed, heading and change in the vertical axis *e.g.* altitude for terrestrial/volant species, and depth for aquatic species, [22], [23], [24] both of which can be recorded using data loggers attached to animals [25]. Changes in either altitude or depth can be determined with high resolution using pressure sensors [26], [27], and heading can now be measured to within 1° [25], [28]. Dead reckoning is undertaken using data-loggers, so devices require recovery in order to access data, although this also means that the efficacy of dead reckoning is not dependant on transmission or reception of data. The strength of dead reckoning is that it produces regular, sequential positional data, in fine resolution without any gaps [21]. Analysis of home range and foraging behaviour of terrestrial animals using GPS telemetry is likely to be biased when habitat types differ in their degree of facilitation of GPS signals [29]. In contrast, the efficacy of dead reckoning is uniform throughout the environment. Ultimately, our ability to describe the adaptive significance of animal movement (e.g. [30], [31]) is reliant on obtaining unbiased, accurate data. This makes the development of terrestrial dead reckoning relevant and significant.

Animal travel speed is an ecologically significant parameter in its own right [31], and has implications for e.g. optimal foraging, food detection and predation risk [30], [32], [33]. Yet speed can be problematic to measure directly. Previously, the speed of terrestrial animals has been measured by manual pursuit of study animals [29] or by estimation from VHF, GPS and satellite telemetry [34], [35], [36]. In fact, GPS telemetry has shown some promise for measuring speed, provided a sufficient sample rate is used [37]. Nevertheless, GPS telemetry becomes less reliable or unworkable in dense vegetation (e.g. forests) or in marine environments. In addition, increasing time between fixes can incur considerable error in speed estimation where constant, straight-line travel between recorded positions is not adhered to. As animals are known to travel tortuous, intermittent paths [38], a more accurate method for measuring speed is required.

The need for an accurate method of estimating speed is pertinent for dead reckoning studies. In this context speed is necessary for estimating the distance travelled in any given direction. Previously this has been done by assuming a constant speed, derived from prior study [39]. This method is likely to incur cumulative errors however, as deviations from this default speed by the animal will displace the estimated position from the actual one [40].

A number of elegant mechanical methods of measuring speed have been proposed for aquatic species, such as propellers [41], [42], [43], [44]; turbines [45], [46]; paddle wheels [47], [48]; and paddles [49] (although current flow can complicate calculations of speed in these environments [40]). In studies of species that undertake terrestrial locomotion, estimating speed is problematic due to the highly variable nature of the environment (*e.g.* wind speed) which rules out the use of mechanical sensors. Suggested options have all been derived from accelerometers (e.g. [50]). Accelerometers are sensors that can be used in animal-attached loggers to measure an animal’s movement and orientation [42] and can even be used to elucidate a wide range of behaviours in free living animals [51], [52], [53]. Stride frequency is readily apparent from acceleration data [54] and generally correlates with stride length, allowing speed to be derived (*c.f.* [55]). This is, however, likely to be subject to substantial variation across body size and species [56], [57]. Another option is assessment of the mean amplitude of acceleration peaks recorded during movement, which has previously been used to estimate energy expenditure in free-swimming sharks [58] although, to our knowledge, this has not been used as a proxy for speed in terrestrial animals. A correlation between speed and amplitude is expected however, as increased stride lengths are expected at higher speeds [59] so further evaluation of this proxy is warranted.

An alternative surrogate measure for speed is Overall Dynamic Body Acceleration (ODBA), which is the sum of the absolute acceleration from all three orthogonal axes (surge, heave, sway) after the static portion of the acceleration signal has been removed see [60]. Put simply, ODBA reflects a combination of acceleration peak frequency and amplitude, and generally correlates well with speed [25], [61]. However Bidder *et al*. [50] found that the relationship between speed and ODBA was subject to variation according to species and gait, much as is stride frequency. In addition, Bidder *et al.* [50] postulated other parameters such as substrate type and incline may further confound matters.

ODBA was originally proposed as a proxy for movement-related metabolic rate [60] and there appear to be good reasons for using this summed quantity in this context [62]. However, acceleration is a vectorial quantity, and its summation for the three axes is likely to over-estimate the physical acceleration experienced by the data logger. Vectorial Dynamic Body Acceleration (VeDBA) uses Pythagoras theorem to calculate the vectorial acceleration, providing values closer to the true physical acceleration experienced. VeDBA also has the added advantage in being insensitive to device orientation, which is not the case with ODBA [58], [62].

This study aims to evaluate metrics derived from tri-axial accelerometers, specifically stride frequency, amplitude of acceleration peaks, ODBA and VeDBA as surrogate measures for speed, particularly examining how much incline and substrate affects them as proxies. Such work is importantfor producing accurate, fine scale measures of speed that can be used in environments which preclude the use of other methods *e.g.* GPS under dense canopy cover [63]. The accuracy of surrogate measures for speed are also important in defining the accuracy of dead-reckoning for free-living terrestrial animals, which may move over very variable terrain. Variability in substrate is difficult to control in test animals, so as with Halsey et al. [61] and Bidder et al. [50], we limited our study to humans, allowing us to test predictions with an easily controlled species.

## Materials and Methods

Acceleration was recorded in three orthogonal axes corresponding to the heave, surge and sway axes of humans (e.g. [62]) using tri-axial accelerometers (8 bit resolution, recording range −3 to 3 *g*; HOBO Pendant G Acceleration Data Logger, Onset Computer Corporation, 470 MacArthur Blvd., Bourne, MA 02532) at sampling rates of 20 Hz (all axes). The devices were placed within a Silastic® (www.thomsonbros.co.uk) saddle to ensure that the logger was held firmly, and strapped in the centre of the back using a cross-chest Silastic harness (see [62] for details).

Experiments were conducted on eight healthy adults (mean age ± SD: 25.55±2.74). The experimental protocol was approved by the ethics committee of Swansea University, and all participants were subject to written informed consent. Whilst equipped with accelerometers, they travelled a defined distance of 10 m, delineated by markers on the ground, at a range of speeds incorporating three different gaits employed by humans; walk, jog and run [64], [65], [66]. During each run, participants were instructed to travel at a constant speed, which necessitated starting the run before passing the first marker and only decelerating once they passed the second. These experiments were conducted on two substrate types; concrete and sand, and 3 incline types; 11° upwards, 11° downwards, and level. The speed of travel was derived via dividing the distance travelled by the time taken to cover the marked course (determined using a stopwatch accurate to 0.01 s). Data corresponding to each run were isolated from the superfluous data, and four metrics derived from the acceleration were calculated.

1. For Overall Dynamic Body Acceleration, all raw acceleration values from each axis were smoothed using a running mean over 2 s [67]. The dynamic acceleration in each of the three axes was calculated for each axis by subtracting the values obtained by the running mean (which constitute the static acceleration [67]) from the raw acceleration values. These dynamic portions of the signal were then converted into absolute positive units and the resultant values from all three channels then summated to give Overall Dynamic Body Acceleration (ODBA, see [60]). Mathematically, this is;

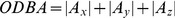
(1)(2) The calculation for Vectorial Dynamic Body Acceleration (VeDBA, see [68]) is similar to that of ODBA, however instead of summating the dynamic acceleration, the vectorial component is derived by;



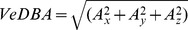
(2)(3) Both Peak Frequency and (4) Amplitude of strides were calculated from the surge axis, as this where the majority of the horizontal acceleration experienced by the tag during locomotion was recorded (discernable from the oscillating wave form produced by the strides during locomotion). Peak Frequency was calculated simply by dividing the number of acceleration peaks during a run by the time taken for run completion. Amplitude was obtained by calculating a mean for the minimum and maximum values recorded during the run (the peaks and troughs of the wave form), and subtracting the minimum from the maximum [58].

The data were subjected to regression analysis to test for a relationship between the metrics and speed. However, since initial inspection of the data showed some bimodality for ODBA according to gait, the data were examined using simple linear regression [61]. The relationships between the metrics and speed were then compared between the 4 substrate/incline conditions using a General Linear Model (GLM): speed ∼ metric + substrate + metric × substrate, using substrate as a fixed factor and the metric as a covariate.

## Results

**Figure 1:**
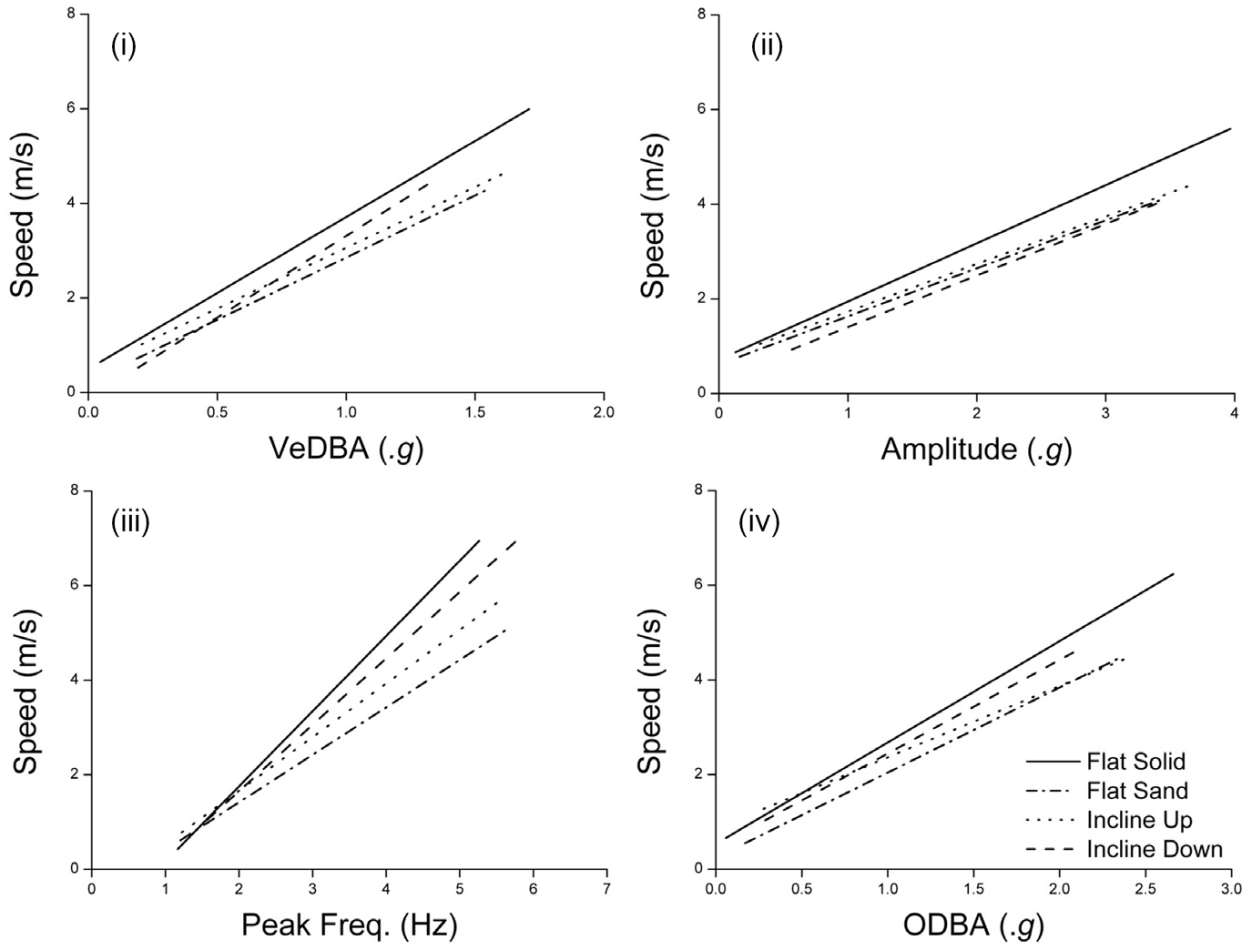
Linear regression between each metric and speed for each substrate/incline. (i) VeDBA, (ii) Amplitude, (iii) Peak Frequency and (iv) ODBA.

For all conditions, there was an approximately linear relationship between the increasing speed and increasing VeDBA, ODBA, peak frequency, and amplitude of acceleration peaks (Fig. 1). The relationship between the metrics and speed appeared to change according to different substrate/incline conditions (Fig. 1) and indeed the GLM showed significant interaction between substrate/incline conditions in each of the metrics (Table 1). Thus, broadly speaking, the relationship between the metrics and speed was not consistent across the various substrates and incline conditions tested.

**Table 1:** Summary of GLM statistics for each metric comparing the relationship with speed under each substrate/incline condition.

Metric	N	df	F	p-value
ODBA	960	4	433.733	<0.001
VeDBA	960	4	545.944	<0.001
Freq	960	4	233.06	<0.001
Amp	960	4	298.744	<0.001

When data for all 4 substrate/incline conditions were collated and the metrics regressed against speed (Table 2), VeDBA was the best predictor of speed (with the highest R^2^ value) with an R^2^ of 0.64, while R^2^ was 0.60, 0.60 and 0.51 for ODBA, stride frequency and amplitude, respectively. Upon regression of the metrics against speed for each of the conditions individually (Table 3), it appears that VeDBA provided the best fit for level concrete and downward slope (R^2^-values of 0.77 & 0.58 respectively), ODBA provided the best fit for level sand (R^2^ of 0.74) and Peak Frequency the best fit for upward slope (R^2^ of 0.69).

**Table 2:** Summary of the statistics for the regression between the metrics (VeDBA, ODBA, Peak Frequency and Amplitude) and speed, with data for all substrates/inclines collated.

Metric	N	p-value	Adj. R^2^	Regression equation
VeDBA	240	<0.001	0.644	= VeDBA*2.964+0.279
ODBA	240	<0.001	0.599	= ODBA*1.907+0.48
Peak Frequency	240	<0.001	0.598	= Freq*1.239-0.826
Amplitude	240	<0.001	0.513	= Amp*1.069+0.628

**Table 3:** Summary of the statistics for the regression between the metrics (VeDBA, ODBA, peak frequency, and amplitude) and speed, with substrate and incline conditions (Level Concrete, Level Sand, Upward Slope and Downward Slope) considered separately.

Metric	Substrate/Incline	N	Adj. R^2^	p-value	Regression equation
	Level Concrete	240	0.763	<0.001	= ODBA*2.14292+0.53462
	Level Sand	240	0.737	<0.001	= ODBA*1.79811+0.24425
ODBA					
	Upward Slope	240	0.483	<0.001	= ODBA*1.50748+0.85617
	DownwardSlope	240	0.427	<0.001	= ODBA*1.97915+0.46534
	Level Concrete	240	0.765	<0.001	= VeDBA*3.21416+0.49782
	Level Sand	240	0.684	<0.001	= VeDBA*2.62951+0.22409
VeDBA					
	Upward Slope	240	0.649	<0.001	= VeDBA*2.57257+0.48899
	DownwardSlope	240	0.577	<0.001	= VeDBA*3.44995-0.1384
	Level Concrete	240	0.680	<0.001	= Freq*1.59084-1.42901
	Level Sand	240	0.727	<0.001	= Freq*1.00487-0.59452
Peak Frequency					
	Upward Slope	240	0.690	<0.001	= Freq*1.13428-0.60751
	DownwardSlope	240	0.570	<0.001	= Freq*1.40467-1.15683
	Level Concrete	240	0.614	<0.001	= Amp*1.23064+0.71234
	Level Sand	240	0.634	<0.001	= Amp*1.01498+0.61474
Amplitude					
	Upward Slope	240	0.560	<0.001	= Amp*1.00469+0.72677
	DownwardSlope	240	0.309	<0.001	= Amp*1.09011+0.31177

## Discussion

### Effect of Substrate on Estimations of Speed

Depending on speed, locomotion on a soft, yielding substrate such as sand requires between 1.15–2.5 times more mechanical work than on harder substrates [69]. This is partly due to the effect soft substrates have on running economy, either by increasing the muscle-tendon work that must be done (during walking), or by decreasing muscle-tendon efficiency (during running). Compliant surfaces such as concrete allow increased energy rebound during locomotion, and this energy return reduces the work required from the runner [70]. On soft substrates this energy conservation is not achieved, so additional work is required, which is expected to be reflected in the dynamic acceleration signal [68]. In light of this, it is little surprising that, at any given speed, values of VeDBA, ODBA and amplitude were all higher over the sand than for the hard substrate (Figure 1), something that can, ultimately, be related to the additional cost of transport over sand.

This additional cost of transport has consequences for estimates of speed. When deriving speed for an animal travelling over a substrate with a higher cost of transport, estimations based on dynamic acceleration will be higher than the true speed. With regard to dead reckoning, this error would increase the estimated distance travelled and displace calculated locations from the true animal locations accordingly.

The effect that the additional cost of transport on soft substrates has on step frequency would result in over-estimation of speed. This should produce similar path problems to those derived using dynamic acceleration (see above). This is because more steps are required on sand per unit distance than on hard substrates (a higher step frequency to reach the same speeds). However, since, for step frequency, the slope component of the regression is most affected by the change in substrate (Figure 1), the error in a dead reckoned track on soft substrate would be reduced (or none) at lower speeds but exacerbated at higher speeds. This is attributable to the dampening capacities of sand, which absorbs some of the stride. Thus, during the stride work is done displacing sand, making take off velocities lower than those of firmer substrates, with the period and distance travelled during strides both being lower [69].

Although this study focuses only on one example of a soft substrate, sand, we expect a perturbation of the relationship between speed and the metrics on any substrate known to incur a different cost of transport or produce a reduction or facilitation in the speed of locomotion. Locomotion on other soft substrates, such as snow, is more energetically demanding than on firm substrates [71], as is locomotion over terrain that may include ‘superstrates’ such as shallow water and dense jungle vegetation [72]. Other experiments have manipulated the compliance of surfaces [28] which result in lower speeds being obtained, so it is reasonable to suggest that surfaces with these properties will also perturb the relationship between dynamic acceleration and speed. Given that some species are particularly likely to encounter numerous substrate types as they move through their environment, workers need to understand the limitations of using fixed, prior calibrations for any of the metrics tested for speed for use in dead-reckoning. Either information on the substrate distribution in the environment and their respective calibrations, or an alternative method for correcting dead-reckoned tracks *ad hoc* is required (see below).

### Effect of Gradient on Estimations of Speed

Much like substrate, locomotion over surfaces of different gradients incurs different costs of locomotion. Compared to locomotion on a flat surface, the metabolic cost of locomotion is higher on positive gradients, and generally lower on negative ones (but greater on gradients >6 degrees, see [24]). This is primarily due to the additional energy needed to overcome gravity on upward gradients, and specifically the mechanical work needed to gain gravitational potential energy. Conversely, on downward gradients, less mechanical work is done as gravitational potential energy is reclaimed to provide propulsion, except on very steep gradients, where work must also be done to resist gravity via active braking [51]. Unlike locomotion on different substrates, where dynamic acceleration, speed and metabolic rate are all correlated, downward grades cause the correlation between metabolic work and acceleration to break down because acceleration can be produced with less mechanical work done by the leg muscles [63].

The most significant effect of gradient on metrics of dynamic acceleration and speed was to increase the variance recorded, particularly for ODBA. VeDBA produced a far higher coefficient of determination than ODBA under the sloped conditions (both upward and downward slope, Table 2). Both metrics are measures of dynamic body acceleration, so this disparity in the coefficients of determination is surprising. Both are calculated from the same raw data output from the tri-axial accelerometer, and static and dynamic acceleration for the three axis are calculated with the same method. In fact, the only difference between the two metrics is the method by which the data for the three orthogonal axes are combined. Where VeDBA is calculated using the vectorial solution to produce the vectorial product of acceleration [68], ODBA is calculated via simple summation of the absolute dynamic acceleration on the three axes [60]. Whilst ODBA provides an easy to use and simple metric, the summation method will inevitably over estimate the proper acceleration. As such ODBA values will always be greater than the corresponding value for VeDBA [62]. However, given that ODBA and VeDBA are so closely correlated, at least during level travel [62], the reason for this difference is not obvious. However, we can conclude that VeDBA would appear the more appropriate metric in environments that may contain frequent changes in surface gradients.

For amplitude the difference is most significant, with R^2^-values for the downward slope being almost half of those for the level concrete (0.31 and 0.61, respectively). Previous research has shown that humans reduce their step length (with which amplitude is associated) when travelling down slopes in order to reduce the friction demand at heel strike, reducing the likelihood of dangerous slips [73]. However, during the present study, participants were seen to adopt both short and long strides depending on the speed attempted. Our study protocol required participants to use a range of speeds, some of which may not have been otherwise attempted. In Sun *et al.* [73], participants were observed walking at self-determined speeds, and so locomotion at higher speeds was not observed so this disparity in stride length responses to the downward slope may have resulted in the particularly low R^2^-values observed.

### Accelerometry and its use as a Proxy for Speed in Dead-reckoning

Bidder *et al*. [50] suggested that as a terrestrial animal moves over various substrate and sloped conditions, the relationship between ODBA and speed would also change and the results from this study support this. This has practical implications for the production of dead reckoned tracks of animal movement because estimations of speed on substrates or gradients that differ from those of the original calibrations will incur error. The same is true for the other metrics tested (Table 3).

Pragmatically, VeDBA would seem the best metric to use overall because it was the strongest predictor of speed when the data from all substrates and gradients were considered together (Table 1). Recent work published by Qasem *et al*. [62] on the merits of using VeDBA over ODBA as a proxy for energy expenditure concluded that there was little practical difference between the two. Indeed, ODBA has become a widely used metric in studies of animal energetics [58], [60], [61], [68], [74], [75], [76]. Clearly though, VeDBA outperforms ODBA as a proxy for speed for species likely to traverse a range of substrates or gradients, except perhaps ’level sand’ conditions where ODBA had a greater coefficient of determination. It may thus be appropriate to use this metric on species that live in environments dominated by this substrate type.

Dead-reckoning over terrain of varying grades might benefit from being informed by GIS (Geographic Information Systems) in some way (e.g. [77]), although GIS information is often limited in scale and may not have the necessary substrate data. It is also possible to derive gradient in quadrupeds from the static acceleration signal because these animals alter their body angle as they negotiate slopes [53], [78]. Similarly, we would expect changes in the form of the acceleration signals for animals moving over different substrates which might usefully inform proxies for speed.

Fortunately, the method of dead-reckoning with corrected speed values proposed by Bidder *et al*. [50], would seem robust enough to deal with any changes in the landscape that might affect the relationship between VeDBA and speed. By this method, estimates of speed are corrected until the calculated tracks accord with ground-truthed positions, obtained via a secondary means [50]. In its crudest sense, this could be the known start and end positions (*i.e.* location of animal release and tag recovery). However, given that the results of the current study show that transitions in substrate and incline gradient are likely to have a significant effect on the dead-reckoned track, periodic ground-truthing would be preferable. This could be achieved simply via deployment of a GPS logger in tandem with the dead-reckoning device, or via VHF telemetry, RFID or animal sightings. In this sense, dead-reckoning could serve to fill the gaps between fixes of less frequent telemetry methods [c.f. 20,40]. Further work is required to determine exactly how frequently such ground-truthing should be undertaken, but the results of this study suggest that it should be more frequent in habitats which are known to contain many types of substrates or gradients. Other issues involved with dead-reckoning, such as heading errors, are yet to be addressed.

### Method Limitations

The coefficient of determination (R^2^) for the regression of (human) speed against ODBA on level concrete is lower than that reported in Halsey *et al.* [61] for comparable conditions. We attribute this to our measurement errors because we derived speed using a stopwatch whereas Halsey *et al.* [61] used a treadmill (*c.f.* [73]). Treadmills provide researchers with a means to define the running speed of participants; however no treadmill is able to emulate the change in substrates required for this study.

Alternative methods for timing runs exist, such as using laser timing gates, and these operate with minimal measurement error. The protocol in the current study utilised the stopwatch because, as far as possible, we standardized measurement protocols, one of which involved travelling over inter-tidal sand where the use of laser gates is not possible. However, given that the present study includes data for 960 runs by 8 individuals, relative differences in coefficients of determination (R^2^) between metrics and substrate/incline conditions are unlikely the result of measurement error.

### Conclusion

When data for all substrate and gradient conditions were collated, VeDBA proved to be the metric with the highest coefficient of determination when regressed with speed. However, relationships between speed and all the metrics tested in the present study were subject to variation due to substrate and gradient. Whilst using prior calibrations of speed to any of the metrics tested may be useful for use in detecting intermittent animal locomotion [38], dead-reckoned tracks produced in this way are likely to produce errors without some secondary means of correction. This is particularly germane in habitats where transitions in substrate and gradient are frequently encountered. These corrections can be conducted via periodic ground-truthing of the dead-reckoned tracks by other methods of telemetry. In this way, dead-reckoning provides a means to obtain fine scale movement data for terrestrial animals without the need for additional data on substrate or gradient.
