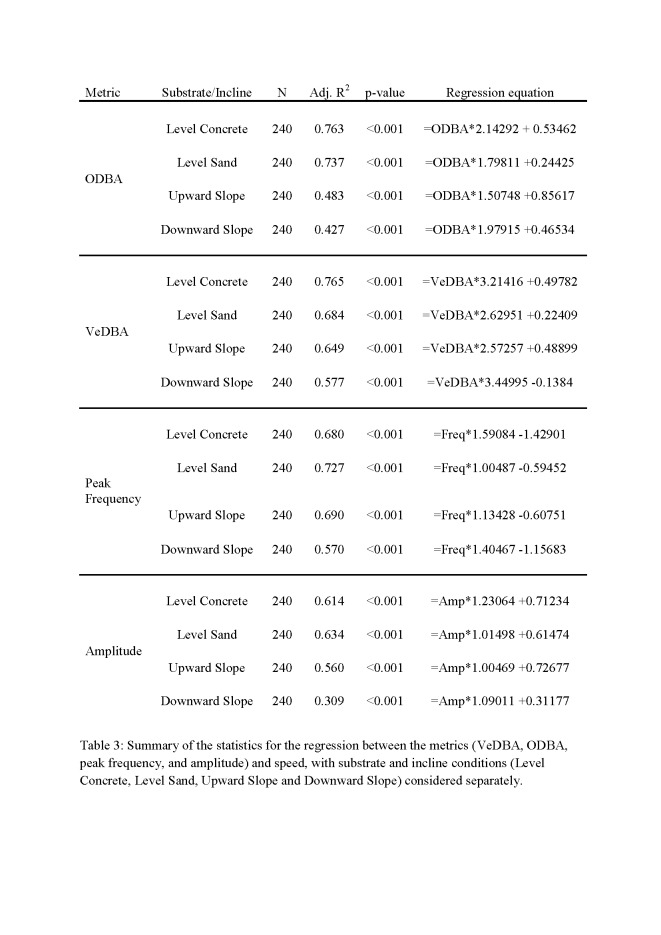# Correction: On Higher Ground: How Well Can Dynamic Body Acceleration Determine Speed in Variable Terrain?

**DOI:** 10.1371/annotation/7715d163-4965-40b1-bc92-68241d9bbe88

**Published:** 2013-11-08

**Authors:** Owen R. Bidder, Lama A. Qasem, Rory P. Wilson

In Table 3, the substrate/incline conditions are not aligned correctly with the corresponding metrics. Please see the corrected Table 3 here: 

**Figure:**