# Correction: On Higher Ground: How Well Can Dynamic Body Acceleration Determine Speed in Variable Terrain?

**DOI:** 10.1371/annotation/cde9d37a-6b33-40d7-b6d4-83cdf443c942

**Published:** 2014-01-23

**Authors:** Owen R. Bidder, Lama A. Qasem, Rory P. Wilson

In the Reference list, references 32-35 and 37 are incorrect. The correction references for 32-35 and 37 are:

32. Vasquez RA, Ebensperger LA, Bozinovic F (2002) The influence of habitat on travel speed, intermittent locomotion, and vigilance in a diurnal rodent. Behavioral Ecology 13: 182-187.

33. Janson CH, Di Bitetti MS (1997) Experimental analysis of food detection in capuchin monkeys: effects of distance, travel speed, and resource size. Behavioral Ecology and Sociobiology 41: 17-24.

34. Laundre JW, Reynolds TD, Knick ST, Ball IJ (1987) Accuracy of daily point relocations in assessing real movement of radio-marked animals. Journal of Wildlife Management 51: 937-940.

35. Morales JM, Haydon DT, Frair J, Holsinger KE, Fryxell JM (2004) Extracting more out of relocation data: Building movement models as mixtures of random walks. Ecology 85: 2436-2445.

37. Witte TH, Wilson AM (2004) Accuracy of non-differential GPS for the determination of speed over ground. Journal of Biomechanics 37: 1891-1898.

